# Long-Term Follow-Up of Significant Improvement After CAPTEM Treatment for Rare Adrenocorticotropin-Producing Cardiac Neuroendocrine Tumor

**DOI:** 10.3389/fendo.2019.00713

**Published:** 2019-10-22

**Authors:** Lin Lu, Qingqing Meng, Xiaoping Xing, Tao Yuan, Huabing Zhang, Naishi Li, Yining Wang, Yuejuan Cheng, Chunmei Bai, Hao Wang, Xin Cheng, Yu Xiao, Boju Pan, Yuan Li, Jian Sun, Zhiyong Liang, Huijuan Zhu, Renzhi Wang, Zhaolin Lu

**Affiliations:** ^1^Key Laboratory of National Health Commission, Department of Endocrinology, Peking Union Medical College, Peking Union Medical College Hospital, Chinese Academy of Medical Science, Beijing, China; ^2^School of Medicine, Tsinghua University, Beijing, China; ^3^Department of Radiology, Peking Union Medical College, Peking Union Medical College Hospital, Chinese Academy of Medical Science, Beijing, China; ^4^Department of Oncology, Peking Union Medical College, Peking Union Medical College Hospital, Chinese Academy of Medical Science, Beijing, China; ^5^Department of Nuclear Medicine, Peking Union Medical College, Peking Union Medical College Hospital, Chinese Academy of Medical Science, Beijing, China; ^6^Department of Pathology, Peking Union Medical College, Peking Union Medical College Hospital, Chinese Academy of Medical Science, Beijing, China; ^7^Department of Neurosurgery, Peking Union Medical College, Peking Union Medical College Hospital, Chinese Academy of Medical Science, Beijing, China

**Keywords:** Cushing syndrome, ACTH syndrome, ectopic, cardiac neoplasms, neuroendocrine tumor, chemotherapy

## Abstract

Ectopic adrenocorticotropic hormone (ACTH) syndrome (EAS) is a rare cause of Cushing syndrome. If routine imaging examinations cannot identify the source of ACTH production, long-term follow-up observation is necessary to determine the etiology. We present the case of a middle-aged male with gradual weight gain and a Cushingoid appearance over 4 years; he provided written informed consent. Laboratory and endocrine tests strongly suggested EAS, although the origin was not detected by multiple imaging methods. Bilateral adrenalectomy was performed to prevent severe complications in the patient. Two and a half years later, a cardiac mass 18 × 23 × 27 mm in size at the junction between the anterior wall of the left ventricle and the middle septum was found together with multiple bone metastases by ^18^F-FDG PET/CT, while the ^68^Ga-DOTATE PET/CT findings were negative. Biopsy of the lumbar vertebrae revealed a neuroendocrine tumor (NET) with positive ACTH staining. The patient underwent chemotherapy by CAPTEM, resulting in shrinkage of the cardiac mass and a significant decrease in the ACTH level. In the case of EAS with an unusual cause, long-term follow-up observation is necessary to determine the source of ACTH production. Cardiac NETs are quite rare in EAS, so treatment selection was also challenging. CAPTEM chemotherapy proved effective in controlling the progression of tumor growth and decreasing the ACTH level in this patient.

## Background

Cushing syndrome (CS) is a rare disease accompanied by hypercortisolemia due to various causes. It is necessary to diagnose CS as early as possible to prevent the occurrence of fatal complications. Pituitary adrenocorticotropic hormone (ACTH)-secreting adenoma and ectopic ACTH syndrome (EAS) account for the etiology of CS in 70% and 10–20% of cases, respectively (1). The most commonly used dexamethasone suppression test (DST) cannot completely distinguish between the two conditions, with a false-negative rate of approximately 20%. Bilateral inferior petrosal sinus sampling (BIPSS) has been considered the gold standard for detecting the origin of ACTH. Bronchial and mediastinal ACTH-producing carcinoid tumors are the most common causes of EAS, representing nearly 90% of all EAS cases. Here, we report a very rare case of EAS caused by a neuroendocrine tumor (NET) that possibly originated in the heart with bone metastasis.

## Case Presentation

The study protocol was approved by the Ethics Committees of the Peking Union Medical College Hospital, Beijing, and written informed consent was obtained from the patient for the publication of this case report.

A 35 year-old Chinese man was admitted to Peking Union Medical College Hospital in April 2012 with complaints of weight gain and a round and red face for 4 years. The patient denied any underlying medical condition, such as hypertension, diabetes and heart disease. There was no significant personal or family history. There were no inherited diseases in his family. His blood pressure was 170/120 mmHg on admission. The laboratory workup showed hypokalemia (serum potassium, 2.5 mmol/l) and elevated morning plasma total cortisol (37.04 μg/dl; normal range, 4–22 μg/dl), morning plasma ACTH (235.0 pg/ml; normal range, 0–46 pg/ml) and 24 h urinary free cortisol (1,136.39 μg; normal range, 12–103 μg) levels. High-dose DST was not suppressible. There were no detectable tumors on dynamic enhanced MRI of the sella or CT of the chest, abdomen or pelvis. BIPSS with the desmopressin stimulation test did not show an ACTH concentration gradient. Therefore, EAS was highly suspected. Further imaging evaluations, including somatostatin receptor scanning, ^18^F-FDG PET/CT and ^68^Ga-DOTATE PET/CT, showed negative findings. Meanwhile, pulmonary infection and hypoxemia aggravated rapidly. Considering the rapid progression of illness and the unclear origin of the ectopic ACTH, bilateral adrenalectomy was performed in August 2013 immediately to improve the life-threatening condition. Subsequently, the patient's general condition significantly improved.

**Figure 1:**
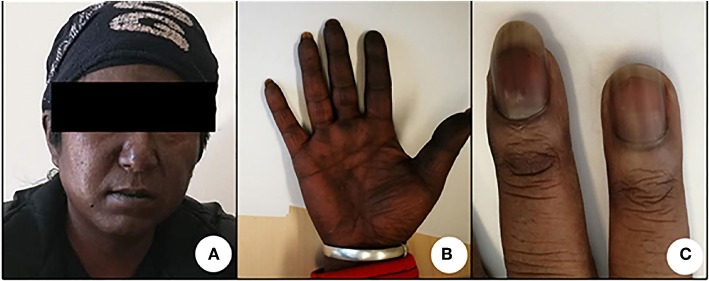
Hyperpigmentation of the patient's skin **(A)**, knuckles and nails **(B)**, and palms **(C)** was photographed in January 2016.

**Figure 2:**
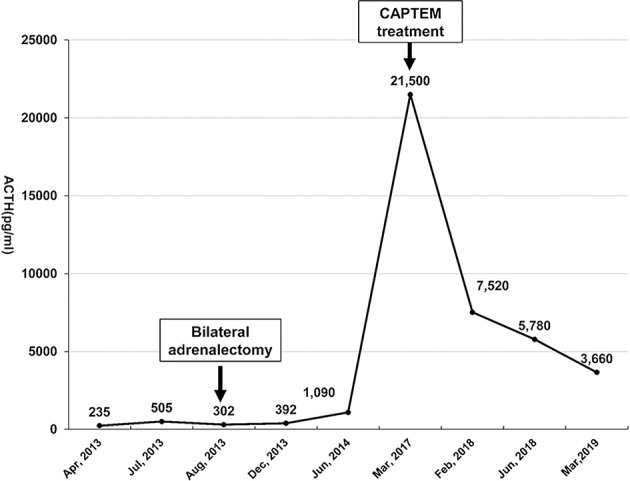
The morning serum ACTH level was monitored from April 2013 to March 2019. Bilateral laparoscopic adrenalectomy was performed in August 2013. Note that his ACTH level increased to over 1,250 pg/mL after January 2015, with the highest level reaching 21,500 pg/ml. However, the ACTH level dropped significantly after the patient underwent CAPTEM treatment. The last follow-up test indicated that the ACTH level dropped to 3,660 pg/ml in March 2019.

**Figure 3:**
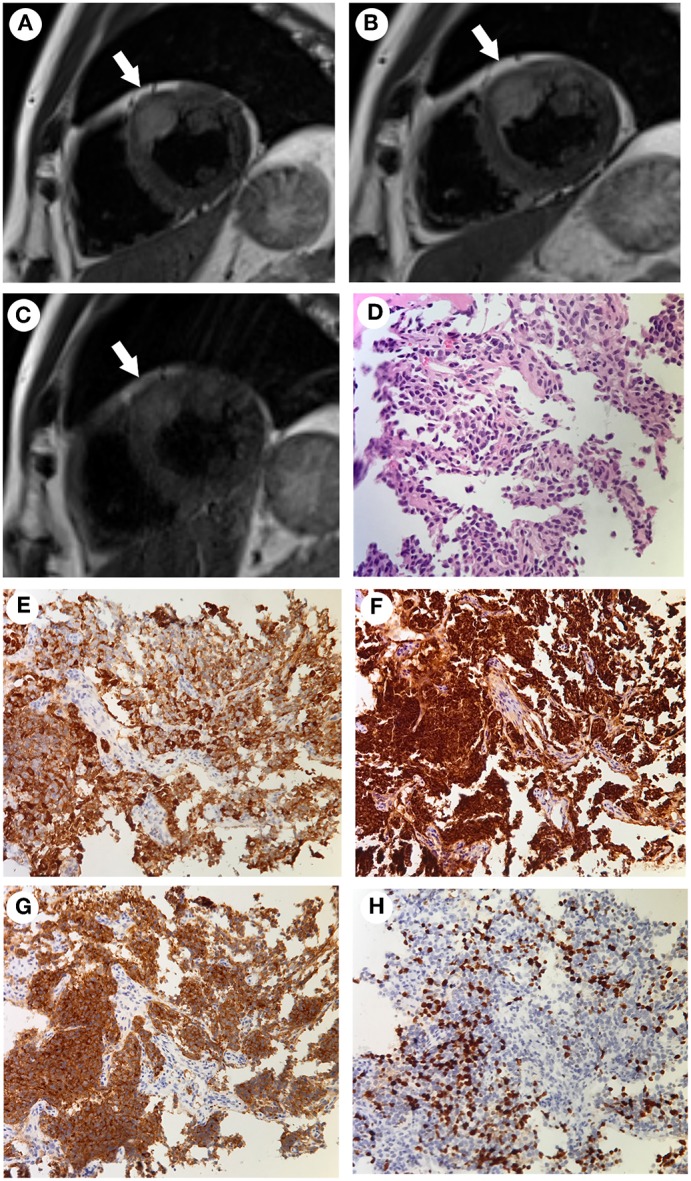
**(A–C)** Cardiac MRI findings acquired at different time points. The tumor was located at the junction between the anterior wall of the left ventricle and the middle septum (arrows). The tumor was 18 × 23 × 27 mm in size when initially found in January 2016 **(A)**. It had increased in size to 20 × 24 × 28 mm in November 2016 **(B)**. After combined chemotherapy with capecitabine/temozolomide for 12 months, the tumor size was 16 × 22 × 22 mm in January 2018 and remained stable to March 2019 **(C)**. **(D–H)** The pathological findings of the bone biopsy of the metastatic vertebrae. Hematoxylin and eosin (HandE) staining showed an invasive tumor cell growth pattern, with abundant eosinophilic cytoplasm and finely granular nuclear chromatin. Mitosis and necrosis were frequent **(D)**. Positive immunohistochemistry staining for ACTH **(E)**, chromogranin A **(F)**, and synaptophysin **(G)**. The Ki-67 index was approximately 20% **(H)** (Magnification, × 200).

The patient participated in regular follow-up examinations annually. His Cushingoid symptoms recovered gradually. The patient's blood pressure and hypokalemia returned to normal. The patient's weight decreased obviously, with disappearance of the roundness and redness of the face. However, his skin color incrementally darkened. Annual chest, abdominal and pelvic CT and somatostatin receptor scanning did not identify any tumors during 2014 and 2015. By the end of 2015, his skin color had darkened to a great extent (Figures 1A–C). His morning ACTH level had increased to over 1,250 pg/ml (Figure 2). In January 2016, although the results of somatostatin receptor scanning were still negative, ^18^F-FDG PET/CT revealed abnormal, high uptake in the anterior wall of the left ventricle near the pericardium, as well as in the sacral region, with osteolytic change. Ultrasonic cardiography and enhanced cardiac CT showed thickening of the aortic root and the left ventricular wall. Furthermore, bone scans revealed abnormal lesions in several regions, including the left occipital bone, right sacroiliac joint, spine and right shoulder joint, indicating multiple bone metastases. Cardiac MRI revealed a mass 18 × 23 × 27 mm in size at the junction between the anterior wall of the left ventricle and the middle septum, involving the adjacent myocardium and pericardium (Figure 3A). This suspicious cardiac lesion was considered a very likely source of ACTH production. However, the patient and family refused cardiac biopsy considering the high risk of the procedure. In November 2016, cardiac MRI revealed a slightly enlarged, irregular cardiac lesion (20 × 24 × 28 mm) at the same location (Figure 3B). Biopsy of the lumbar vertebrae was performed, and the pathological results showed neuroendocrine tumor (NET) infiltration in the bone and bone marrow. The tumor cells were well-differentiated, and mitosis and necrosis were frequent (Figure 3D). The tumor cells showed positive staining for ACTH, chromogranin A and synaptophysin (Figures 3E–G), as well as cytokeratin. The Ki-67 index was 20% (Figure 3H). Since the patient had no evidence of detectable lesions apart from the cardiac mass and multiple bone metastases after long-term follow-up observation, it was strongly suspected that his cardiac lesion was a primary ACTH-secreting, high-grade NET.

The challenging location of the cardiac lesion made surgical resection very risky. After consultation with a multidisciplinary team, it was decided to treat the patient with combined oral chemotherapy with a CAPTEM (capecitabine/temozolomide) regimen (orally taking capecitabine 2.5 g/day on the 1st to 14th day and temozolomide 300 mg/d on the 10th to 15th day of each month, followed by discontinuation until the next cycle). Beginning in March 2017 to March 2019, he underwent 24 cycles of chemotherapy without any evidence of further progression of the cardiac lesion (Figure 3C). During the periods medication was administered, he developed the side effects of mild bloating, nausea, and vomiting, which could be tolerated without medical treatment. The above symptoms improved during the periods medication was not administered. There were no other side effects, such as skin rash, bone marrow inhibition and abnormal liver function and renal function. Cardiac MRI indicated shrinkage of the cardiac lesion to 16 × 22 × 22 mm after 8 cycles of chemotherapy, and the bone scan indicated stable conditions. In addition, the ACTH level dropped from 21,500 pg/ml (before CAPTEM treatment) to 3,660 pg/ml. Both the image and hormone test results indicated a partial response to CAPTEM chemotherapy.

## Discussion

EAS is the most challenging etiology in the management of CS. Among tumors in EAS, the prevalent types are bronchial carcinoid tumors (5–40%), thymic carcinoid tumors (5–42%), small cell lung carcinoma (3.3–50%), pancreatic carcinoid tumors (7.5–25%) and pheochromocytoma (2.5–25%) (1, 2). Based on previous publications, no cases of EAS due to a cardiac NET, either primary or metastatic, have been reported thus far.

Cardiac NETs are rarely documented, and most of them are metastatic. According to the current literature, few cases of primary cardiac NETs have been reported (3), and nearly all reported metastatic cases have origins in the gastrointestinal tract, lungs or mediastinum (4). It is noteworthy that cardiac paragangliomas (PGLs), which could arise from paraganglia located at the base of the heart and atria, are another common type of cardiac endocrine tumor (5, 6). However, cardiac NETs and PGLs can be differentiated under the microscope by the main components of PGLs, including clusters of chief cells and characteristic sustentacular cells (7). In addition, the positive staining for cytokeratin in this case ruled out the diagnosis of PGL. In the present case, metastatic high grade NETs with positive ACTH staining were confirmed by pathology. Furthermore, there was no evidence of the primary origin of the metastatic bone NETs other than the cardiac lesion after 6 years of follow-up observation. Therefore, a primary cardiac ACTH-secreting NET and a metastatic cardiac NET of unknown origin were considered highly as possibilities.

During imaging examinations to detect the EAS source, the cardiac lesion was not detected until 2.5 years after adrenalectomy by ^18^F-FDG PET/CT. Therefore, the lack of sensitive imaging methods for the early detection of cardiac NETs may have resulted in a delayed diagnosis. It is not uncommon that some ectopic ACTH-producing NETs are such latent tumors that are quite difficult to detect during the onset of the disease. It is speculated that the deficiency of negative feedback to ACTH production by ectopic NETs after bilateral adrenalectomy may promote tumor proliferation. On the other hand, the previously occult lesion gradually progressed and could be detected by imaging examinations as the disease duration increased. Considering the special location of the lesion, surgery was quite risky for this patient. In recent years, the CAPTEM regimen has been shown to be highly effective and well-tolerated for the management of both well-differentiated and metastatic NETs (8–10). Finally, the CAPTEM regimen was administered to the patient. After this treatment, some shrinkage of the cardiac mass was observed, and the ACTH level dropped 83.0%. Thus, CAPTEM treatment had a beneficial effect on the patient.

In conclusion, cardiac ACTH-producing NETs, either primary or metastatic, are extremely rare. In the beginning, with combined imaging methods, including CT, MRI, somatostatin receptor scanning and PET/CT, the exact location of the cardiac NET remained undetected, leading to a delay in the diagnosis until multiple bone metastases had occurred. After nearly 24 cycles of chemotherapy with the CAPTEM regimen, slight shrinkage of the cardiac mass, no progression of the metastatic bone lesions and significant improvement in the ACTH level were observed. Additionally, the patient tolerated the treatment well. Thus, considering the possibility of disease progression and other distant metastases, lifelong follow-up observation is needed to monitor the disease in this case.

## Ethics Statement

The study protocol was approved by the Ethics Committees of the Peking Union Medical College Hospital, Beijing, and the participant provided written informed consent.

## Author Contributions

LL, QM, XX, CB, YC, and YW carried out the studies, participated in collecting the data, and drafted the manuscript. YX, JS, BP, YL, and ZLi participated in the pathological research. TY, HZha, NL, XC, and HW performed the statistical analysis and participated in its design. HZhu, RW, and ZLu helped to draft the manuscript. All authors have read and approved the final manuscript.

### Conflict of Interest

The authors declare that the research was conducted in the absence of any commercial or financial relationships that could be construed as a potential conflict of interest.
